# GO-Based Membranes for Desalination

**DOI:** 10.3390/membranes13020220

**Published:** 2023-02-10

**Authors:** Rui Ge, Teng Huo, Zhongyong Gao, Jiding Li, Xia Zhan

**Affiliations:** 1 Key Laboratory of Cleaner Production and Integrated Resource Utilization of China National Light Industry, Beijing Technology and Business University, Beijing 100048, China; 2 The State Key Laboratory of Chemical Engineering, Department of Chemical Engineering, Tsinghua University, Beijing 100084, China

**Keywords:** graphene oxide, desalination, interlayer spacing, longitudinal mass transfer pathways, wrinkles

## Abstract

Graphene oxide (GO), owing to its atomic thickness and tunable physicochemical properties, exhibits fascinating properties in membrane separation fields, especially in water treatment applications (due to unimpeded permeation of water through graphene-based membranes). Particularly, GO-based membranes used for desalination via pervaporation or nanofiltration have been widely investigated with respect to membrane design and preparation. However, the precise construction of transport pathways, facile fabrication of large-area GO-based membranes (GOMs), and robust stability in desalination applications are the main challenges restricting the industrial application of GOMs. This review summarizes the challenges and recent research and development of GOMs with respect to preparation methods, the regulation of GOM mass transfer pathways, desalination performance, and mass transport mechanisms. The review aims to provide an overview of the precise regulation methods of the horizontal and longitudinal mass transfer channels of GOMs, including GO reduction, interlayer cross-linking, intercalation with cations, polymers, or inorganic particles, etc., to clarify the relationship between the microstructure and desalination performance, which may provide some new insight regarding the structural design of high-performance GOMs. Based on the above analysis, the future and development of GOMs are proposed.

## 1. Introduction

With the rapid growth of the global population and industrial activity, water environment issues are affecting the sustainability of modern industry and society, and water shortages have become one of the most urgent issues that needs to be addressed [1,2,3]. According to a World Resources Institute (WRI) study, humans are expected to use 56% more water than could be sustainably used by 2030. Currently, about 1% of the world’s fresh water comes from desalination, a source that can provide more than 300 million people (about 4% of the global population) with their daily water needs [4]. Desalination plays a vital role in the present and future as the demand for fresh water increases and climate problems become more severe [5,6,7]. Currently, desalination technologies are mainly divided into two categories: thermal-based desalination and membrane-based desalination. Thermal-based desalination separates water from non-volatile contaminants such as salt using thermally driven phase changes, such as multi-effect distillation (MDE) and multi-stage flash distillation (MSF). Membrane-based desalination relies on external pressure to drive water molecules through semi-permeable membranes to separate them from salt, which is achieved through reverse osmosis (RO) or nanofiltration (NF). Compared to thermal-based desalination, membrane-based desalination achieves the target screening capacity under relatively mild conditions with lower energy consumption. In recent years, membrane-based desalination was considered to be one of the most economical and effective methods to afford clean water due to its energy efficiency, high efficiency, simplicity of operation, and ease of scale, resulting in this form of desalination accounting for 70% of global desalination capacity [8,9]. Traditional membrane-based desalination technologies include RO and NF. RO is still the most commonly used desalination technology due to its operational simplicity and high energy efficiency, but the process is a highly energy-intensive process, requiring between 2 and 4 kWh for the production of 1 m^3^ of clean water [10]. Additionally, RO effluent treatment is expensive [11,12]. Nanofiltration is a pressure-driven membrane separation technique that allows for selective interception, through dimensional screening, of polyvalent and univalent ions, as well as small molecules with different molecular weights or different charge properties [13]. NF is widely used in desalination, wastewater reclamation, and industrial substance separation because of its low cost, high efficiency, low energy consumption, and simplicity of operation [14]. Nanofiltration membranes can remove ions, salinity, and macromolecules from water but are susceptible to contamination and constrained by the trade-off effect, which greatly hinders the ability to produce high-quality water. Compared to RO and NF, pervaporation (PV) desalination technology combines the characteristics of thermal-based and membrane-based desalination and has many significant advantages, such as high desalination efficiency, the fact it is not limited by salt concentration or osmotic pressure, good antifouling performance, etc. [15]. In PV, the driving force is the chemical potential gradient. When atmospheric pressure is applied to the feed side of the membrane, negative pressure is generated by applying a purge gas or vacuum to the permeable side. The solution–diffusion model is used to achieve seawater desalination [16]. PV has excellent rejection (>99.5%) and low energy consumption (~2 kWh/m^3^). PV membrane materials usually use hydrophilic polymers, and long-term operational stability can be achieved using appropriate coatings; however, existing permeation vaporization membranes have low porosity and complex tortuous mass transfer channels, resulting in low fluxes and inadequate rejection rates [17].

Membrane materials with high permselectivity are the heart of membrane separation technology [18]. Two-dimensional graphene oxide (GO), rich in oxygen-containing groups on lamellar planes and edges, maintains excellent hydrophilicity and solubility in aqueous solution, which achieves preferential adsorption of water molecules and provides the possibility of membrane preparation under mild conditions. The 2D nanochannels formed by the stacking of GO nanosheets can enable ultrafast transport of water molecules and hinder the passage of ions [19]. The degree of oxidation of GO is usually 40–60% [20] and the thickness of a single layer of GO is approximately 1 nm, with lateral dimensions of the layer in the micron range [21]. GO nanosheets are self-assembled through stacking, which forms GO membranes, and single-layer GO nanosheets include oxidized and non-oxidized regions. The oxygen-containing functional groups separate adjacent GO nanosheets from each other to form interlayer channels in laminar flow membranes, which also facilitate the passage of water through GO nanosheets in a hydrated state. The non-oxidizing regions provide a fast network of capillaries that the associated water transports through with almost frictionless flow [22,23]. Nanochannel dimensions are usually influenced by ambient humidity, with interlayer spacing ranging from 0.5 to 1.4 nm (the interlayer spacing of GOMs increases in wet states due to the embedding of water molecules) [24]. The adjacent GO nanosheets are interconnected via hydrogen bonding and π-π interaction; however, as the ionization of carboxyl groups on the layers makes the layers negatively charged, there is a strong electrostatic repulsion between adjacent layers, and the presence of oxygen-containing functional groups ensures the GO nanosheets have good hydrophilic properties. Therefore, the GO membranes formed by stacking swell after water absorption and are easily destroyed and dispersed [25]. GO membranes can be produced as free-standing membranes or supported by porous substrates as composite membranes [26]. Free-standing membranes typically require greater thickness to satisfy sufficient strength, but the increased thickness significantly reduces the permeability of the membranes [27]. Therefore, ultrathin GO membranes with support layers are the main focus of current research.

GO provides good hydrophilicity and membrane forming properties while also exhibiting poor swelling resistance and membrane stability. There are three main typical phenomena that occur in desalination application due to GOM instability. First, in aqueous solution, the hydration between the hydrophilic functional groups on GO and the water molecules causes repulsion between the GO layers and increases interlayer spacing. GOMs are prone to decomposition or redispersion in aqueous solution and lose separation capability completely. Second, the non-oxidized zones of GOMs lack the support of oxygen-containing functional groups and are prone to collapse during pressurization tests, which reduces layer spacing in the non-oxidized zone region and decreases water permeation flux [28]. Third, constrained by the “trade-off” effect, it is difficult to improve the separation performance and permeation flux of GOMs at the same time.

During the permeation of water molecules across GOMs, molecules first enter the edges or defects of GO nanosheets through longitudinal mass transfer channels before then entering the GO interlayer and sliding through the interplanar 2D interlayer transport channels. Up to now, researchers have conducted extensive research into regulation of the microstructure of GOMs, such as regulation of the interlayer spacing of GOMs through various methods so that water molecules could pass through while the entry of salt ions was hindered. Additionally, the stability of GOMs was enhanced after interlayer spacing was fixed, and the longitudinal mass transfer channels of GOMs could also be regulated by decorating or constructing channels so that water molecules could pass through GOMs quickly and improve permeation flux. Therefore, precise construction of mass transfer channels and the stability of the use process are the keys to improving the separation performance and permeation flux of GOMs [22].

**Figure 1:**
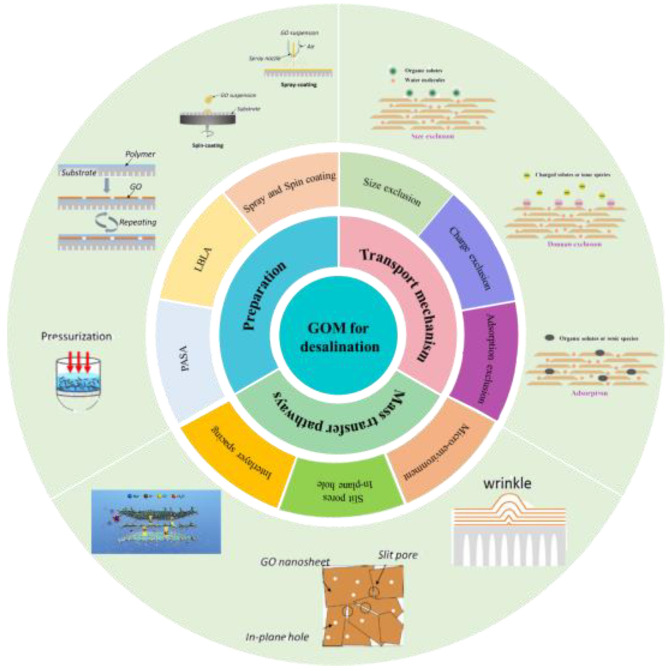
The framework of this review. Figures have been reproduced from [29,30,31,32,33] with permission of Elsevier.

This review aims to summarize the available methods for regulating the microstructure of GOMs, including regulation of GOM interlayer spacing, regulation of longitudinal mass transfer channels, and regulation of GO sheets layer wrinkles, as shown in Figure 1. Additionally, the structure of GO, mass transfer mechanisms, and GOM preparation methods are briefly introduced. Finally, the progress of GOM research and applications is summarized, which provides new ideas for the development of a new generation of high performance GOMs.

## 2. Preparation Methods of GO Membranes

The oxygen-containing functional groups on GO planes and edges endow GO with good hydrophilicity and solubility in water. GO lamellar membranes were usually prepared by vacuum/evaporation/pressure-assisted self-assembly (VAS, EAS, and PAS); layer-by-layer assembly (LBL); coating, spraying, and spin-coating methods; and the dehydration process. The macro/microstructure and separation performance of GOMs is significantly influenced by preparation methods. The structure of the GO membranes can be regulated by various factors, including GO concentration, driving forces, deposition cycles, spraying/spinning rate, time, etc. This subsection summarizes the preparation methods of GOMs and discusses the advantages of different methods in practical applications.

### 2.1. Pressure-Assisted Self-Assembly (PAS)

**Figure 2:**
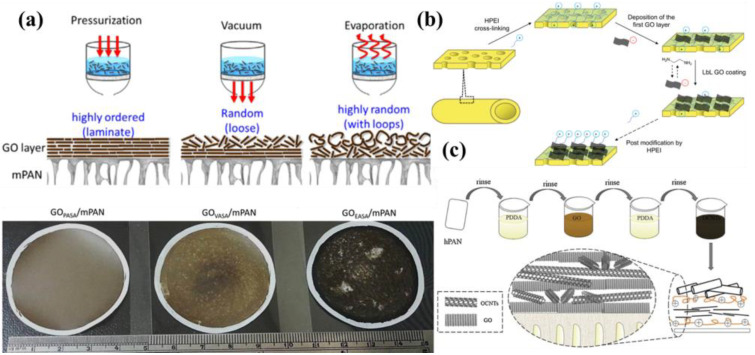
(**a**) Schematic diagrams of GOM preparation by VAS, EAS, and PAS and the physical diagram of a GOM are shown from left to right. Reproduced from [29] with permission of Elsevier, 2015. (**b**) Preparation of GOMs using the LBL method with the GO and EDA deposition cycle. Reproduced from [35] with permission of Elsevier, 2016. (**c**) GO and OCNTs alternately assemble to form a sandwich-structured GOM. Reproduced from [36] with permission of Elsevier, 2018.

Vacuum/evaporation/pressure-assisted self-assembly (VAS, EAS, and PAS) is widely used for the preparation of GOMs [34]. The GO suspension is deposited and filtered through a porous substrate (usually UF or MF) to form a laminar flow structure of a thin selective layer of GO nanosheets, which is shown in Figure 2a. Tsou et al. investigated the effects of three assembly methods on GO film structure: (1) In the deposition of GO by PAS, the coverage of the GO layer was ordered. (2) For VAS, as cake thickness increased or filtration rate decreased with time, cake resistance increased and vacuum level decreased, causing the GO layer near the substrate to become dense; however, the GO layer away from the substrate becomes loose. (3) Deposition of GO by EAS was influenced by the upward driving forces associated with the vaporizing liquid; the order of GO assembly layers was random and the surface was therefore very rough [29]. It was also found that GOMs prepared by PAS had excellent separation performance due to the dense and highly ordered laminate structure of GO.

Different driving forces in the self-assembly process lead to different microstructures in the GO assembly layer. The change in driving force magnitude and direction had a significant effect on the structure of the GO assembly layer.

### 2.2. Layer-by-Layer Assembly (LBLA)

The layer-by-layer (LBL) self-assembly method is usually used to produce polyelectrolyte membranes at the molecular level through the assembly of polyelectrolytes with opposite charges [37]. GO is an ideal candidate for the preparation of LBL membranes due to its 2D lamellar structure and deprotonated carboxyl groups of GO in solutions.

Before the LBL process, the support layer is usually modified to form a charged surface. Chung et al. obtained a highly efficient GO separation layer by cross-linking the substrate layer with hyperbranched polyethyleneimine (HPEI) and then immersing the membrane in GO and ethylenediamine (EDA) solutions alternatively with different deposition cycles (Figure 2b) [35]. Similarly, Nan et al. prepared highly positively charged nanofiltration (NF) membranes using graphene oxide (GO) and polyethyleneimine (PEI) using the LBL self-assembly method [38]. The high aspect ratio and unique two-dimensional structure of GO nanosheets allows them to be easily assembled on the membrane surface, and the fast water transport channels within the GO nanosheets ensure high water flux. GO membranes prepared using the LBL method can provide high repellency to salt ions of different chemical valence by controlling the nature of the charge deposited on the surface of the membrane layer. LBL self-assembly methods that assemble two nanomaterials into multilayer structures are also available. Xu et al. assembled GO and oxidized carbon nanotubes (OCNTs) alternately to form sandwich-structured GOMs (resulting in adjusted interlayer spacing in GOMs), which were subsequently cross-linked using polyelectrolytes; the obtained GOMs exhibited significantly improved surface roughness and permeability (Figure 2c) [36].

In general, the thickness of the GOM is increased by increasing the number of assembling cycles, deposition time, or the concentration of GO solution, which generally increases the mass transfer resistance and leads to reduced fluxes of GO membranes.

### 2.3. Spray and Spin Coating

Spraying is the vertical spraying of the suspension onto the substrate under controlled conditions (spraying time, suspension concentration, functionalized graphene, membrane drying) to obtain a membrane with mechanical strength [39]. Spin coating involves applying the membrane casting solution to the spin substrate in a spin-coating instrument and relies on centrifugal action to spread the cast film on the substrate to obtain a functional layer of uniform thickness.

Nair et al. prepared GO membranes using spray and spin coating. SEM and X-ray analyses showed that this GOM had a clear layered structure consisting of crystals several microns long, and interlayer distance d was about 10 Å [40]. Kim and colleagues systematically investigated two spin-coating methods of preparing GOMs: (1) contacting the surface of the support membrane with the gas–liquid interface of the GO solution and then spin coating it to prepare a multilayer GOM and (2) rotational casting of the GO solution on the membrane surface to prepare GOMs [41,42]. The GO deposition in method (1) was controlled by electrostatic and hydrophilic interactions between the GO nanosheets and the polymer membrane. In alkaline aqueous solution, the ionization of carboxylic acid groups at the edges of GO sheets was negatively charged; therefore, the edges of GO sheets repelled each other, resulting in the uneven deposition of GO sheets. In contrast, GO solution–membrane contact in method (2) contributed to tighter GO deposition due to the face-to-face attraction between the GO sheets, which overcame the edge-to-edge repulsion of the GO sheets.

### 2.4. Dehydration

One of the methods for preparation of GO membranes/foams is the stacking of GO sheets through the use of a dehydration process. Akhavan et al. [43] loaded a GO suspension into flexible PET glass and dried it for 24 h under ultraviolet light from a fluorescent UV lamp at 80 °C. The deposited GO sheets were then cut into squares. The thickness of the GOF layer was measured with a micrometer to be approximately 15–50 μm.

The micro-structure of the GOM prepared with different preparation methods varies significantly, which determines the physi-/chemical properties and separation performance of GOM in practical application. Thus, the optimum preparation method should be chosen according to the separation system and separation requirement.

## 3. Transport Mechanism of Small Molecules in GOMs

**Figure 3:**
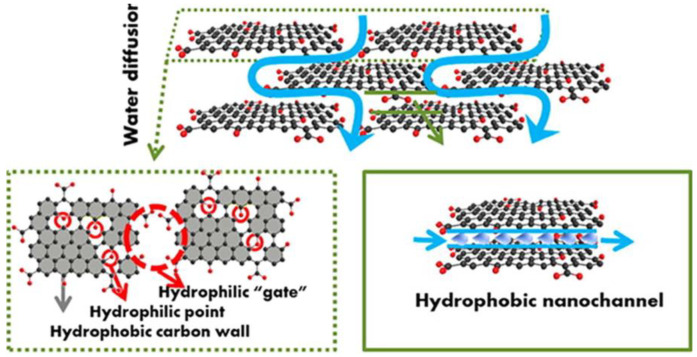
Mass transfer of water molecules in a GOM. Reproduced from [45] with permission of American Chemical Society, 2016.

It is usually considered that water molecules first go to the hydrophilic “gate” (space between edges of two adjacent GO nanosheets or defects of GO) for aggregation and then slip through into hydrophobic 2D nanochannels, as shown in Figure 3. The GO lamellae have oxidation and non-oxidation zones. According to the slip theory, water molecules show frictionless super-speed flow in the non-oxidation zone of the GO layer, and the water permeation rate is very fast [28]. The oxidation zone with abundant oxygen-containing functional groups allows for strong hydrogen bonding interactions with water molecules [29], which hinders the rapid slip of water molecules; however, GOMs can be chemically modified through a chemical reaction with oxygen-containing functional groups. Nair et al. found that submicron-thick GOMs can absolutely impede the flow of liquid and gas molecules, allowing only the penetration of water vapor; this is probably attributed to the presence of a gap between the non-oxidized GO sheets, which allows for the transport of water vapor through low-friction channels [38]. Based on isotope labeling, it was demonstrated that liquid water can permeate at ultrafast speeds through millimeter-long nanocapillaries in GO membranes [44]. The abundant oxygen-containing groups at the edges and defects of GO layers have good hydrophilicity, which could allow water molecules to preferentially enter the two-dimensional channels between GO layers and quickly slip through into the non-oxidized regions of GO layers; thus, GOMs can be used for ultrafast water permeation [45].

**Figure 4:**
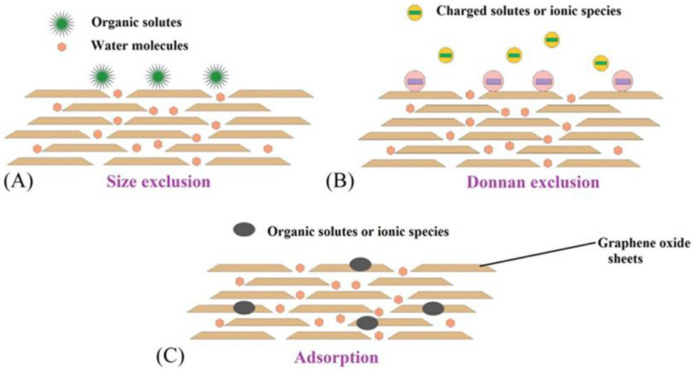
Diagram of the separation mechanism of GOM (**A**) size exclusion, (**B**) charge exclusion, and (**C**) adsorption exclusion. Reproduced from [31] with permission of Elsevier, 2019.

The salt exclusion mechanism may be mainly due to physical sieving, Donnan exclusion, and ion adsorption on GO nanosheets by cation–p interactions, p–p interactions, or the coordination of metal species [45]. Figure 4 shows a diagram of the separation mechanism of GOMs. Based on a size-exclusion mechanism, large ionic species or bulky molecules are filtered due to the presence of smaller nanochannels in GO membranes. Studies have shown that the size of the nanochannels can be increased or decreased by manipulating interlayer spacing between GO sheets to precisely separate ions or bulky molecules [31]. Moreover, GO membranes with larger nanochannels than the hydration radius of ions also showed good ion rejection. Therefore, it was considered that GOMs can also be separated by the Donnan effect exclusion process in addition to size exclusion [38,46]. Considering that the hydration radii of ions such as SO_4_^2−^(3.79 Å), Cl^−^(3.32 Å), and Na^+^ (3.58 Å) are slightly smaller than the hydration radii of carbon nanochannels (formed by two adjacent GO nanosheets) (about 3.98 Å), the effect of physical sieving on salt rejection may be limited [45].GO membranes generally show negative charge due to the proton of carboxyl groups at the edge or tip of the GO sheet, which makes GO membranes reject negatively charged organic species or divalent ions [47]. However, the charge on the GO membrane can be changed. The Donnan exclusion mechanism is often used to explain the rejection properties of charged nanofiltration membranes. According to Donnan exclusion, the ion concentration on the membrane surface is not equal to the ion concentration in the bulk solution. The membrane surface has a higher concentration of counter-ions (ions with opposite charges) than the bulk solution. There is a low concentration of co-ions (ions with the same charge as the membrane) on the membrane surface. When external pressure is applied to the membrane, water molecules can pass through the membrane, while anions are rejected due to Donnan exclusion [45]. Besides, significant rejection can be achieved by strong adsorption of small ion species through various interactions with different regions on the GO sheets [48]. Metal cations can form coordination interactions with any oxygen-containing functional groups on the GOM, blocking the nano-capillary channels responsible for metal cation penetration and restricting the passage of metal cations, thus exerting a rejection effect [47].

## 4. Regulation of GOM Mass Transfer Pathways

For GOMs, the mass transfer channels are mainly divided into three categories: (1) two-dimensional interlayer channels formed between GO nanosheets parallel to each other; (2) longitudinal mass transfer channels generated through edge stacking or defects of GO nanosheets; and (3) longitudinal mass transfer channels provided by the surface pores of GO nanosheets. Small molecules pass through the longitudinal mass transfer channels and diffuse into the two-dimensional interlayer channels of the GOM. For ultrathin membranes composed of single- and few-layer GO nanosheets, longitudinal mass transfer channels consisting of GO nanosheet edges and their surface pores are the main transport pathways that play a key role in size rejection; for membranes composed of multiple GO nanosheets forming selective layers, longer interlayer nanochannels between parallel GO nanosheets provide selectivity and determine permeation. Precise regulation of GOM mass transfer pathways is crucial when improving the separation performance of GOMs. Here, the discussion focuses on summarizing the construction and separation performance of transverse and longitudinal mass transfer channels in multilayer GO membranes.

### 4.1. Regulation of Interlayer Spacing

For the multilayer stacked structure of GOMs, separation performance mainly depends on the size of interlayer spacing, and the precise regulation of interlayer spacing has a significant effect on molecular transport efficiency. Since GO is rich in oxygen-containing functional groups (carboxyl, hydroxyl, epoxy groups, etc.), it easily adsorbs water molecules, which leads to membrane swelling and increases interlayer spacing. This makes the separation performance of small molecules weaker, and interlayer spacing must be reduced to ensure the separation selectivity of the membrane. However, the reduction in interlayer spacing tends to lead to a decrease in permeability [49]. Therefore, precise adjustment of the size of the two-dimensional mass transfer channels between GO layers is significant regarding simultaneous improvement of the selectivity and permeability of the membrane and mitigation of the trade-off effect. In this section, some methods for modulating interlayer spacing that have been reported in the literature are presented. These methods mainly include (1) reduction, (2) cation-π interaction, (3) covalent cross-linking, (4) polymer intercalation, and (5) inorganic particle intercalation, and the influence laws of various modulation methods on the desalination performance of GOMs are analyzed and discussed based on these methods.

#### 4.1.1. Reduction of Graphene Oxide

Generally, it is believed that GOMs with tight interlayer structures will have better separation selectivity as the reduction can remove the oxygen-containing functional groups on the surface and edges of GO, thus reducing interlayer spacing and improving separation selectivity. The reduction of GO can be achieved by thermal reduction [50], chemical reduction [51], physical reduction [52], green reduction [53], and biological reduction [54]. In addition, the reduction treatment of GO can increase the proportion of non-oxidized regions on GO lamellae and can weaken the electrostatic repulsion between the lamellae while improving the stability of the membrane. Simultaneously, it will also reduce the hydrogen bonding network between oxygen-containing functional groups, as well as bonding between oxygen-containing functional groups and water, which is conducive to the rapid flow of water molecules between the layers and improves water permeability.

**Figure 5:**
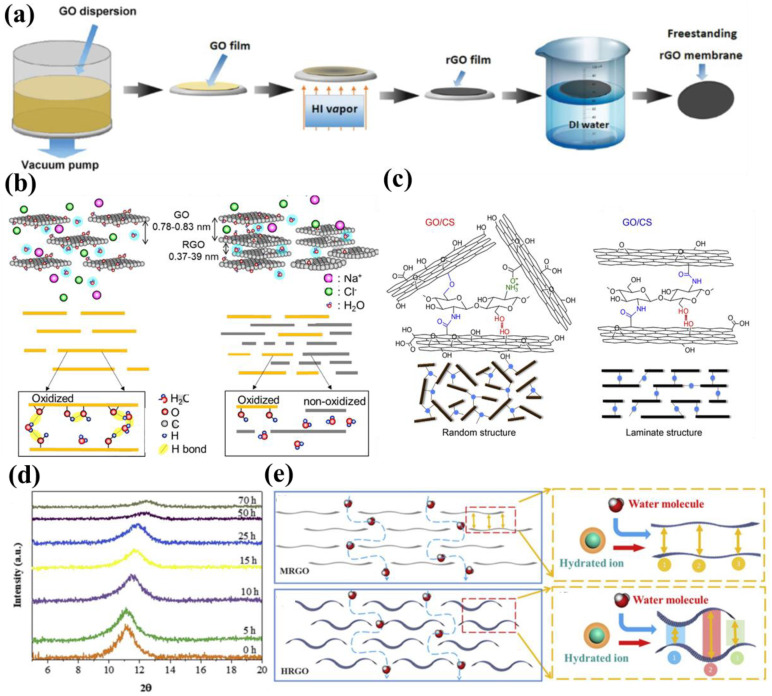
(**a**) Schematic diagram of the fabrication of a freestanding rGO membrane using the HI steam reduction method. Reproduced from [55] with permission of Wiley, 2015. (**b**) Water molecule transport in GOMs and rGO membranes. Reproduced from [23] with permission of Elsevier, 2019. (**c**) Structure schematic of the rGO/CS membrane, GO/CS membrane. Reproduced from [56] with permission of Elsevier, 2017. (**d**) XRD pattern of rGO membrane interlayer spacing with reduction time. Reproduced from [57] with permission of Elsevier, 2020. (**e**) Schematic diagram of HRGO and MRGO mass transfer channels and corresponding XRD. Reproduced from [57] with permission of Elsevier, 2020.

As shown in Figure 5a, freestanding ultrathin rGO membranes with a thickness of 20~40 nm were prepared by hydriodic acid (HI) vapor chemical reduction of GO membranes and water-assisted stratification. This chemical reduction strategy using HI steam was relatively simple and efficient. After HI reduction, interlayer spacing was reduced from 0.87 nm in pristine GO membranes to 0.35 nm in rGO membranes due to the removal of oxygen-containing groups on the GO surface [55]. It is important to note that vapor exposure time plays a crucial role in the degree of reduction in GO laminates. Although the chemical reduction of GO membranes using chemical reagents can be performed in just a few minutes, the use of strong acid vapor is not very environmentally friendly. In contrast, a gentler reduction can be achieved through thermal treatment [22].

The thermo-reduction method was often used to prepare rGO membranes as a low-energy, low-pollution, and environmentally friendly reduction method. Huang et al. prepared rGO membranes with different reduction times (from 0.5 h to 8 h) by heating the GO dispersions for reduction at 160 °C [23]. It was found that the physicochemical properties of rGO layers and the membrane formation characteristics were highly dependent on thermal reduction time. During the reduction process, the longer the reduction time, the more oxygen-containing functional groups were removed, the more non-oxidized zones, the less opportunity to establish hydrogen bonding networks, and the more easily water molecules passed quickly from the non-oxidized zones (Figure 5b). Hung et al. dispensed GO suspensions at 90 °C for different periods of hydrothermal reduction (0, 6, 12, 24, 48, and 72 h) and then added the rGO suspensions to CS (chitosan) solutions to form rGO membranes. Hydrothermal reduction removed most of the carboxyl groups from GO, reducing the tendency of ionic complexation between rGO and CS, thus providing enough time for the formation of a layered ordered stacking structure during drying of the rGO/CS membranes that creates efficient water channels between the nanosheets (Figure 5c) [56]. Li et al. reduced GOMs in air at 80 °C and 150 °C and found that the mild heat treatment produced relatively more uniform 2D separation channels than the high heat treatment [57]. The XRD images showed that GOM interlayer spacing became narrower with the increase in thermal reduction time (Figure 5d). In addition, peak intensity kept decreasing because the functional groups were removed, which resulted in defects on the GO surface. HRGO (high-temperature reduction) had a complex laminar flow structure inside the membrane, and the limiting factor for water/ion transport during permeation was only zone 1. Comparatively, the channel size inside the MRGO (mild annealing) membrane was relatively more uniform. Thus, there was transport resistance to both water and salt ions throughout the channel (Figure 5e), which indicated that reduction of the GO membrane was also affected by the reduction temperature. The rGO membrane separation performance obtained by mild reduction was also better. Guan et al. dispersed a certain amount of GO into deionized water via ultrasonication and centrifugation and obtained a black rGO suspension using a hydrothermal reduction of GO [58]. The prepared rGO and GO membranes were characterized, and it was found in XRD that rGO showed a broad peak near 2θ = 25°, while GO showed a sharp peak at 2θ = 11°. According to Bragg’s law, rGO had narrower interlayer spacing compared to GO. The reduction degree of GOMs was intimately related to thermal reduction temperature and time.

In summary, reduction methods can reduce the oxygen-containing functional groups on GOMs, which can thus reduce GOM interlayer spacing and effectively hinder hydrated ions with larger ion diameters while significantly improving separation performance. The increased non-oxidation zone can also reduce the agglomeration of GO nanosheets and polymers during the preparation of GOMs. There are also some new processes to reduce GO, including a solar-mediated method [59], electrochemical method [60], photocatalytic method [61], biomolecule-assisted method [62], hydrogen nanobubble-rich solutions [63], catalytic method [64], and reduction by light-driven proton pump molecules [65]. Although great progress has been achieved regarding reductions in interlayer spacing in regulated GOMs, a significant reduction in oxygen functional groups in the reduced GOM may lead to a reduction in hydrophilicity and permeability; thus, there are studies that have added cations, MOF, etc., to the rGO membranes to stabilize interlayer spacing and membrane structure while increasing mass transfer channels, thus improving the flux of GOMs.

#### 4.1.2. Cation Intercalation

**Figure 6:**
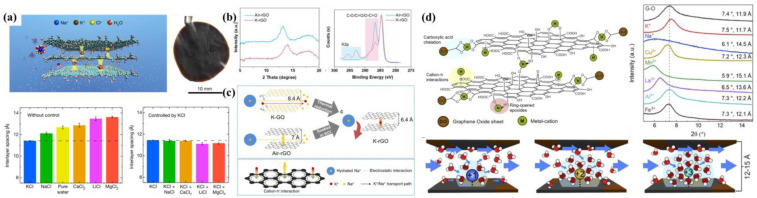
(**a**) Schematic diagram of K^+^ fixed interlayer spacing in a GOM, a GOM prepared by GO suspension, and interlayer spacing of a GOM immersed in pure water or a salt solution. Reproduced from [32] with permission of NPG, 2017. (**b**) XRD and XPS patterns of K-rGO membranes. Reproduced from [68] with permission of Elsevier, 2021. (**c**) Schematic diagram of Na^+^ permeation in K-GO, Air-rGO, and K-rGO interlayer channels. Reproduced from [68] with permission of Elsevier, 2021. (**d**) The adsorption process of cations and GOMs, XRD of a GOM modified by different cations, and the effect of different valence cations on interlayer spacing and water flow. Reproduced from [69] with permission of Elsevier, 2020.

Studies have shown that the effect of cations on GO interlayer spacing is mainly due to strong non-covalent cation-π interactions between cations and aromatic rings on GO sheets, as well as interactions between hydrated cations and oxygen-containing functional groups, which could regulate interlayer spacing and thus exclude salt ions while inhibiting the swelling of GOMs in water. Chen et al. used K^+^, Na^+^, Ca^2+^, Li^+^, and Mg^2+^ for precise control of the interlayer spacing of GOMs (Figure 6a) and were able to control the variable range of interlayer spacing to within 1 Å [32]. They immersed the GOMs in pure water or various salt solutions; GOM interlayer spacing in pure water was 12.8 ± 0.2 Å [66,67], while the interlayer spacing of GOMs in salt solutions was 11.4 ± 0.1 Å, 12.1 ± 0.2 Å, 12.9 ± 0.2å, 13.5 ± 0.2 Å and 13.6 ± 0.1 Å and ranked from wide to narrow in the order of MgCl_2_ > LiCl > CaCl_2_ > pure water > NaCl > KCl.

Cations not only existed in terms of cation-π interactions with the aromatic ring on GOMs to adjust the interlayer spacing of GOMs, but also promoted the reduction of GOMs. Yuan et al. added K^+^ to rGO membranes, which resulted in a stable and narrow interlayer channel due to the strong K^+^-π interaction, thus enabling a K-rGO membrane with high rejection of NaCl to be obtained [68]. It could be seen in the XRD that the interlayer spacing of the K-rGO membrane was significantly narrower after thermal reduction (Figure 6b). Reportedly, K^+^ improved the alignment of GO lamellar structures and may thus increase the thermal conductivity of GO layers [70,71]. Therefore, K^+^ could promote the thermal reduction of GOMs, leading to a higher degree of reduction coupled with narrower interlayer spacing.

The GOM interlayer spacing intercalated by different valence cations varies significantly, which may result in different micro-structures of GOM and physi-/chemical micro-environment of mass transfer pathways. Ching et al. examined the effects of monovalent (K^+^ and Na^+^), divalent (Cu^2+^ and Mn^2+^), and trivalent (Al^3+^, Fe^3+^ and La^3+^) cations on GOMs in PV [69]. As revealed from the XRD in Figure 6c, the cation-modified GOM interlayer spacings were ordered from wide to narrow as follows: Mn^2+^-GO>Na^+^-GO>La^3+^-GO>Cu^2+^-GO>Al^3+^-GO>Fe^3+^-GO>GO>K^+^-GO. Interlayer spacing exhibited this phenomenon because GO membranes modified with cations that have low electronegativity and a large hydration radius will significantly swell when in contact with water. Besides hydration radius, the electronegativity of the cation itself also influences the interlayer spacing and stability of GOMs. GOM interlayer spacing in water was between 12 and 15 Å, which can accommodate two to four layers of water molecules (the influence of different valence cations on water flow through the interlayer spacing can be seen in Figure 6c). After thermal treatment, the K-rGO membrane maintained high and stable NaCl rejection of about 90% over 19 days. The results demonstrated that the K-rGO membrane exhibits good efficiency and stability in selective separation of NaCl from water.

The above studies show that cation-π interactions can successfully modulate GO interlayer spacing, thus achieving improved permeation flux and separation performance. However, compared to amines cross-linking GO, metal cations have no significant effect on the physicochemical properties (e.g., hydrophilicity, functional group species, and surface structure) of the membranes when connecting GO lamellae. Metal cations continue to face many problems when used as crosslinkers to join GO sheet layers due to their own limitations (cross-linking ability, ion size, etc.): (1) The insufficient ability of monovalent cations (Na^+^) to link GO lamellae, especially in an aqueous environment, making it difficult for the cross-linked GOMs to maintain a stable structure. (2) Although the strong coordination ability of multivalent metal cations (Fe^3+^, La^3+^, etc.) can effectively cross-link GO, the interlayer spacing of the cross-linked membrane is limited by the size of the cross-linked cations and the regulation of interlayer spacing can only be carried out in a small range, making it difficult to prepare GOMs with large interlayer spacing and high permeability. (3) The metal cations embedded in the interlayer of GOMs are easily detached from the interlayer by ions exchanged with hydrochloric acid or other monovalent metal cations (Na^+^, Li^+^, etc.), leading to decreased membrane stability [72]. Future research for cationic cross-linked GO membranes should focus on the interaction strength between metal ions and GO and combine these findings with theoretical calculations to explore the synergistic effect of metal ions and other cross-linking agents to obtain high-performance GOMs.

#### 4.1.3. Covalent Cross-Linking

The deprotonation of carboxyl groups on GO layers in aqueous solution usually causes the GO layers to be negatively charged, which results in electrostatic repulsion between neighboring GO layers. It has been demonstrated that pure GOMs disperse rapidly in water, a drawback that greatly limits the practical application of GOMs, whereas the use of a series of amines as cross-linkers (including aliphatic and aromatic amines) can effectively link GO lamellae and enhance the stability of the membrane. In addition, differently structured cross-linker molecules allow for the construction of different interlayer spacings to meet different separation needs.

**Figure 7:**
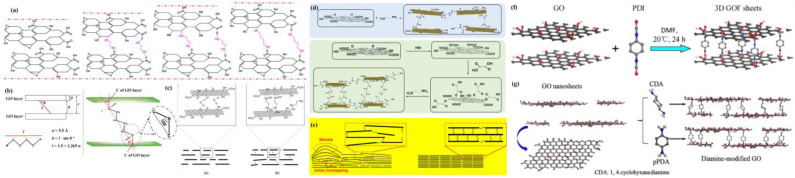
(**a**) Structural diagrams of GO, A2-GO, A3-GO, and A4-GO. Reproduced from [73] with permission of Elsevier, 2019. (**b**) Schematic diagram of the theoretical calculation of Ax-GO interlayer spacing. Reproduced from [73] with permission of Elsevier, 2019. (**c**) Schematic diagram of EDA cross-linked in parallel or adjacent to GO nanosheets. Reproduced from [48] with permission of ACS, 2015. (**d**) Reactions involved in membrane preparation of GO/EDA and OAGO/EDA. Reproduced from [74] permission of Elsevier, 2018. (**e**) Schematic diagram of the microstructure of GO/EDA and OAGO/EDA membranes. Reproduced from [74] permission of Elsevier, 2018. (**f**) Cross-linking of PDI and GO to form a GOF. Reproduced from [75] permission of Elsevier, 2016. (**g**) Schematic diagram of cross-linking GO with CDA and Ppda. Reproduced from [76] permission of Elsevier, 2018.

Acylation reactions occur between the amino group on the amine and the carboxyl group on the GO, and nucleophilic addition reactions occur with the epoxy group. The acylation reaction forms amide bonds (-CONH-) and nucleophilic addition reaction induces ring opening of the epoxy group to form C-N bonds. The covalent bond generated by the reaction can firmly cross-link the amine and GO. Qian et al. selected a series of aliphatic terminal diamines with different kinetic diameters, such as 1,2-diaminoethane (A_2_) and 1,3-diaminopropane (A_3_), as the embedded molecules of GO to regulate and control the interlayer spacing of GO, which increased as the size of the cross-linked molecules increased (Figure 7a) [73]. They investigated the relationship between GO interlayer spacing and aliphatic terminal diamine chain length (C_n_H_2n_(NH_2_)_2_(Figure 7b)) and the relationship between interlayer spacing and water flux. By inserting molecules into the GO layer and adjusting the effective pore size of the GO membrane to make it less than or equal to the minimum size of the hydrated ions, the optimal value for GO layer spacing can be determined. This study provides guidance for the selection of amines for precisely regulating GO interlayer spacing.

Cross-linking of amines occurs not only due to face–face interactions in GO nanosheets, but also at the edges of GO nanosheets that contain a large number of oxygen-containing groups capable of undergoing acylation with amines. Thus, edge–edge and edge–face cross-linking in GO nanosheets can also occurred, which affects the transfer channels of GOMs. Zhang et al. used ethylenediamine (EDA) to cross-link two parallel or adjacent GO nanosheets (Figure 7c). For parallel GO nanosheets, cross-linking with EDA reduced the interlayer spacing of GO, increased the rejection rate, and reduced water permeability. For adjacent GO nanosheets, connection with EDA might change their longitudinal mass transfer channels [48]. The method of changing the GO longitudinal mass transfer channels will be described in the next section. Lin et al. grafted oxalic acid (OA) molecules on the GO base, modified GO (OAGO) with abundant COOH groups at both the edge and the base, and subsequently cross-linked with EDA to prepare GOMs via pressurized filtration, thus achieving modulation of the interlayer spacing (interlayer spacing <1.0 nm) of GOMs (Figure 7d) [74]. Figure 7e shows that the cross-linking of diamine and GO resulted in a regular and orderly stacking of GO nanosheets and a reduction in wrinkles on the GOM, which could limit the swelling of the GOM and improve the stability of the membrane due to the attachment of EDA to the GO nanosheets.

Due to the numerous oxygen-containing functional groups of GO nanosheets, including hydroxyl and carboxyl groups at the edge of the sheet and carbonyl and epoxy groups on the surface of the sheet, GO can be easily crosslinked with aromatic amines [77,78], isocyanates [79], and acyl chlorides [80] to form a three-dimensional graphene oxide framework (GOF). This can improve the flexibility and hydrophilicity of GOMs. It is also suggested that the spacing between layers should be enlarged to improve the performance of GO. Isocyanate can react with the hydroxyl and carboxyl groups of GO to form carbamate and amide functional groups, and through the connection of isocyanate, there can be a solid covalent bond between adjacent GO nanosheets. Feng et al. used 1,4-phenylene diisocyanate (PDI) and GO cross-linking to modulate interlayer spacing [75], which increased due to the support of PDI between the layers; aromatic diamines had excellent mechanical stability, and cross-linking with GO nanosheets could modulate interlayer spacing while also improving the stability of GOMs (Figure 7f). Qian et al. cross-linked GO nanosheets with two diamine molecules as cross-linkers (1,4-cyclodiamine (CDA) and p-phenylenediamine (pPDA)) [76] to moderate GO interlayer spacing (Figure 7g). After cross-linking with CDA and pPDA, the corresponding interlayer spacing of the GOM was 9.09 Å and 11.29 Å, respectively, which was larger than that of the original GOM (8.65 Å) [81]. Therefore, aromatic diamines were inserted into the GO lamellae to increase the interlayer spacing of GO, which thus improved the water flux and stability of GOMs.

Amines can effectively regulate GO interlayer spacing and stabilize the structure of GOMs. Thus, amines have become the most widely studied substances for GO cross-linking. However, the addition of amines complicates the process. Moreover, most of amines are highly toxic and can be harmful to the experimenters and the environment if not handled properly. In addition, the cross-linking of amines and GO mainly relies on the acylation and nucleophilic addition reactions between the amino groups and the oxygen-containing functional groups on GO to generate covalent bonds, which results in the massive depletion of epoxy functional groups that thereby reduces the hydrophilic and anti-pollution ability of GOMs. At present, the sequence of acylation reaction and nucleophilic addition reaction between amines and GO oxygen-containing functional groups is unclear, and further investigation is needed to explore these reactions. It is thought that the development of environmentally friendly cross-linking agents and green and efficient cross-linking methods will probably be the highlight of future research on GOM cross-linking.

#### 4.1.4. Polymer Intercalation

**Figure 8:**
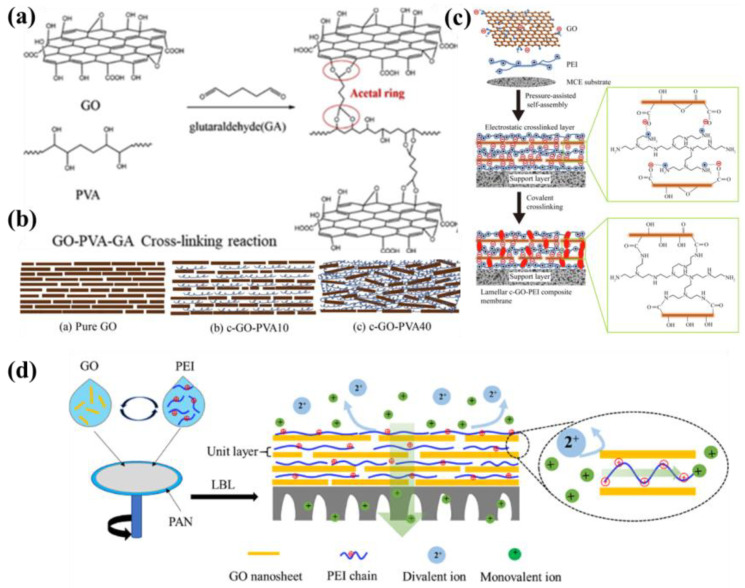
(**a**) PVA intercalated GO under the linkage of the cross-linker GA. Reproduced from [84] permission of Elsevier, 2020. (**b**) Brick and mortar model of a GO layer in a PVA-intercalated GOM Reproduced from [84] permission of Elsevier, 2020. (**c**) Schematic diagram of the process for PEI-cross-linked GO nanosheets. Reproduced from [19] permission of Elsevier, 2022. (**d**) LBL method used to prepare a PVA-intercalated GOM. Reproduced from [85] permission of Elsevier, 2020.

Under the action of cross-linking agents, the polymer insert in the GO layer can adjust interlayer spacing, optimize the mass transfer channel of the membranes, and improve the water flux of the membranes. It can also be used as a linker to fix the GO layers and stabilize the structure of GOMs. Polyvinyl alcohol (PVA) is rich in hydroxyl groups, is easily cross-linked into a mesh structure, has favorable biocompatibility, and exhibits stable physicochemical properties [82,83]. Sun et al. intercalated PVA in GO through the cross-linker GA (Figure 8a) to illustrate the effect of PVA intercalation on the structure of the GO layer in a brick–mortar model, where GO was the brick, PVA was the mortar, and GA was the binder [84]. As illustrated in Figure 8b, the original GOM featured a highly ordered orientation, and GO interlayer spacing was progressively expanded after intercalation with flexible PVA. When PVA content was low, the interlayer spacing of GO gradually expanded with the increase in PVA content. However, when PVA was excessive, the PVA chains would interconnect, agglomerate, and accumulate between GO layers, destroying the regular accumulation state of GO layers. When the content of PVA reached 10 %, mass transfer reached its highest level due to the synergistic effect of proper and favorable interlayer spacing and structurally ordered mass transfer channels.

Polyethyleneimine (PEI) is one of the most commonly used polymers for regulating the interlayer structure of GOMs because of its abundant amino group, excellent hydrophilicity, and large positive charge, and its special long-chain structure allows for the simultaneous attachment and fixation of multiple GO nanosheets. Wang et al. used PEI-cross-linked GO nanosheets of different molecular weights and adjusted the interlayer spacing of the layered GO membranes to desalinate hypersaline through permeation vaporization (PV) [19]. Between PEI and GO, the electrostatic interaction and covalent bonding formed a double cross-linked structure, which effectively limited the dissolution of GO and allowed for interlayer spacing to be adjusted through adjustments to the molecular weight of PEI and conversion of the surface charge of the GOM to a positive charge, thus repelling ions (Figure 8c). The membrane cross-linked with larger PEI (c-GO-PEI 70k) exhibited the strongest structure and excellent permeation vapor desalination performance, with water flux of 86.0 kg/m^2^ h and 99.99% desalination rate at 90 °C for a 10 wt% NaCl salt solution. Huang et al. introduced positively charged polyethyleneimine (PEI) into negatively charged GO interlayers using layer-by-layer assembly (LBL) to separate monovalent/divalent ions through the synergistic effect of particle size sieving and electrostatic repulsion (Figure 8d) [85]. PEI introduction also controlled the charge properties of the interlayer channels and increased the hydrophilicity of the membrane surface. The regular interlayer channels of GOMs enlarge due to the charge interaction of GO with PEI and the filling of PEI. At present, numerous studies have been conducted on polymer intercalation in GOMs using various polymers. Chitosan (CS) is a naturally environmentally friendly polymer with good hydrophilicity. Hung et al. first reduced GO to obtain rGO membranes to remove most of the carboxylate ions that prevented ionic complexation between the negatively charged carboxylate ions of GO and the positively charged protonated amines of CS (-COO-H_3_^+^N-R), resulting in extreme membrane aggregation. They then inserted hydrophilic CS molecular chains between the rGO laminates, which improved the dispersion of GO and adjusted its interlayer spacing so that rGO/CS would stack and self-assemble into laminar structures [56].

GOMs themselves are limited by the “trade-off” effect and fail to achieve a simultaneous increase in total flux and separation factor; therefore, their performance still has great capacity for improvement. By intercalating the polymer between the GOM layers, the GO interlayer structure is regulated. A polymer with excellent hydrophilicity can preferentially adsorb water molecules to increase the flux of GOMs and exclude other particles with large hydration diameters. However, owing to the poor compatibility between GO and polymers, the prepared membranes are prone to non-selective defects, which results in the reduced selectivity of the membranes. Therefore, enabling better compatibility between GO and polymers will be one of the key research directions for GOMs and organic–inorganic composite membranes in the future.

#### 4.1.5. Inorganic Particle Intercalation

The two-dimensional mass transfer channels of GOMs are long and tortuous, and regular and tight channels weaken water molecule permeability. The smaller channel size at the collapse between GO layers without the support of oxygen-containing functional groups makes it difficult for water molecules to pass through. The currently adopted solution for enhancing the permeability of lamellar membranes is to intercalate nano-sized spacers between the layers, such as TiO_2_ [86], MXene [87], and metal–organic frameworks [88], which can accomplish remarkable improvements in GOM flux and separation capacity.

**Figure 9:**
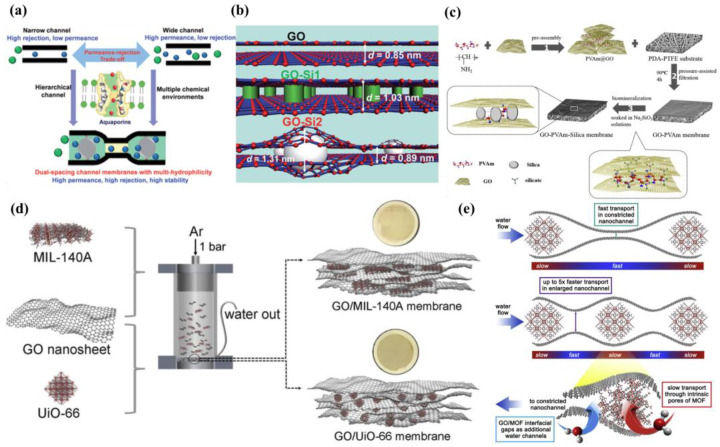
(**a**) Design of alternating double-spaced channels in 2D material membranes to overcome trade-off effects. Reproduced from [89] permission of RSC, 2019.(**b**) Schematic diagram of differently structured GOMs. Reproduced from [89] permission of RSC, 2019. (**c**) Schematic diagram of preparing a GO-PVAm-Silica membrane. Reproduced from [90] permission of Elsevier, 2020. (**d**) Schematic diagram of a prepared GO/MOF composite membrane and a composite membrane structure. Reproduced from [91] permission of Elsevier, 2019. (**e**) Schematic diagram of water transport for different loading amounts of MOF nanoparticles embedded in GO nanochannels and the interfacial gap between MOF nanoparticles and GO nanosheets. Reproduced from [91] permission of Elsevier, 2019.

Wang et al. proposed the in situ generation of silica nanoparticles (SiO_2_) in specific regions between GO layers to achieve the fabrication of GOMs with alternating double-spaced channels (Figure 9a) [89]. The hydrophilic SiO_2_ nanoparticles locally widened the interlayer channel and improved water permeability. In the alternating nanoparticle-free regions, GO layer bending and π-π interaction preserved the narrow hydrophobic channels and promoted high solute rejection (Figure 9b). In addition, SiO_2_ acted as a cross-linking agent, which gave the GOM superior stability in organic solvents. It was arduous to obtain high-performance GOMs by cross-linking single nanoparticles. Pan et al. prepared a GO-PVAm-Silica membrane, as shown in Figure 9c, where they first cross-linked GOM with polyvinylamine (PVAm) to obtain stable mechanical properties, and PVAm then induced bionitrosis to generate SiO2 in situ as a second cross-linker to further fix the size of the two-dimensional mass transfer channels [90]. GO-PVAm-Silica membranes displayed higher swelling resistance than GO-PVAm membranes, with a fixed interlayer spacing of about 0.62 nm in water, high permeability, unique sieving properties for hydrated ions, high NaCl rejection above 99.99%, and 80.2 ± 0.8 kg/(m^2^h) total permeation flux when treating 3.5 wt% NaCl solution at 70 °C. In addition, the GO-PVAm-Silica membranes maintained excellent operational stability over the 168 h test.

Metal organic frameworks (MOFs) are new class of crystalline materials with flexible nanostructures whose tunable pore structures and chemical functions have attracted the attention of researchers. In the past few decades, MOFs have been extensively used in catalysis [92], energy storage [93], gas separation [94], and nanomedicine [95]. MOFs have also shown excellent separation properties for various small molecules [96,97]. Compared to other conventional nanomaterials, MOFs possess highly homogeneous and tunable pore structures and have great potential to become effective intercalation spacers in sheet GOMs due to their ability to alleviate the constraints of “trade-off” effects. Sui et al. prepared GO/MOF composite membranes by inserting two MOFs with different structures (i.e., MIL-140A and UiO-66) into laminated GOMs using a pressure-assisted filtration method (Figure 9d) [91]. When a small amount of the MOF was used (MOF to GO weight ratio below 0.3), water permeability decreased slightly; however, when a larger amount of MOF was added (weight ratio of 0.5), water permeability increased significantly by 92% compared to GOMs, with the increase in water permeability attributed to an increase in average interlayer spacing between GO nanosheets. Small amounts of MOF particles impede water transport in GOM nanochannels, resulting in a lower water permeability coefficient; large amounts of MOF particles increase GO interlayer spacing and form very fast water transport channels. In addition, interfacial gaps between the non-smooth MOF nanoparticles and GO nanosheets were created as additional water transport channels to provide higher water permeability (Figure 9e).

Inorganic particles usually have good mechanical stability, and the insertion between GO layers could play a favorable supporting role that is unaffected by the solvent, thus increasing stability and the range of practical applications for GOMs. However, there are some urgent problems related to inorganic particle intercalation. For example, (1) when inorganic particles grow in situ between GO layers, it is difficult to precisely control their growth direction and quantity, which easily causes the uneven growth of inorganic particles and results in zigzag channels between GO layers that make it difficult for water molecules to pass through; and (2) when MOF particles are intercalated into the GO layers, they tend to agglomerate between the GO layers, destroying the two-dimensional mass transfer channels and hindering the rapid transport of water molecules. Moreover, the preparation process of MOF particles is often complicated, the experimental operation is relatively dangerous, the preparation cost is high, and it is not easy to mass produce. Future research may focus more on precisely controlling the in situ growth of inorganic particles on GO nanosheets, ensuring MOFs are evenly distributed between GO layers, and taking advantage of the special advantages of inorganic particles intercalated with GOMs to obtain ultra-high-performance GOMs.

### 4.2. Regulation of Longitudinal Mass Transfer Channels

The longitudinal mass transfer channel of GO membranes can be adjusted from the following two aspects: one involves decorating longitudinal mass transfer channels to ensure the integrity of the layered structure and improve separation selectivity, and the other involves the construction of longitudinal mass transfer channels by drilling holes on GO lamellae to achieve efficient and accurate screening.

#### 4.2.1. Decorating the Longitudinal Mass Transfer Channel of the GO Nanosheets

GOM has ultra-high permeability, but due to its good hydrophilicity it tends to expand in aqueous solution, resulting in structural instability and lack of selectivity, both of which are highly affected by the microstructure of GOMs. The irregular interlayer accumulation of GO results in the longitudinal orientation of microporous defects. These microporous defects are a key limiting factor controlling the selectivity of GOM solutions. GOMs with fewer or smaller microporous defects will have stronger selectivity.

**Figure 10:**
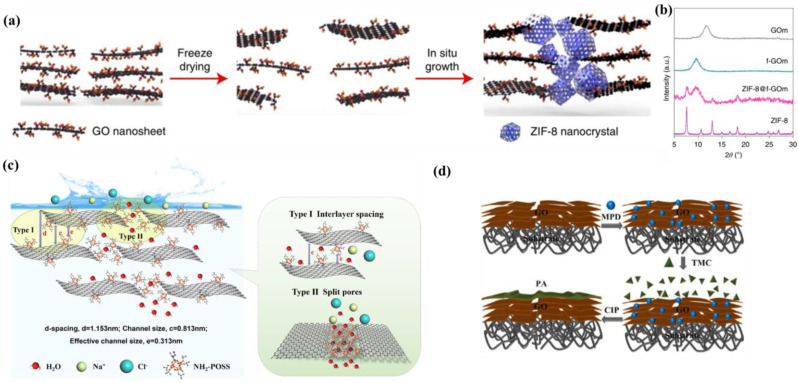
(**a**) Schematic of the preparation of ZIF-8@f-GOm, where the GO layer was first freeze-dried and ZIF-8 nanocrystals were then grown in the microporous defects. Reproduced from [98] permission of NPG, 2021. (**b**) XRD patterns of GOm, f-GOm, ZIF-8@f-GOm, and ZIF-8. Reproduced from [98] permission of NPG, 2021. (**c**) Schematic of the water ion transport mechanism in POSS@GO membranes. Reproduced from [15] permission of Elsevier, 2022. (**d**) Formation of the PA-GO membrane by confined interfacial polymerization. Reproduced from [99] permission of Elsevier, 2018.

The longitudinal mass transfer channel defects were compensated for by filling them with inorganic particles. Zhang et al. prepared a ZIF-8 nanocrystalline hybrid frozen GOM (ZIF-8@f-GOM) in which the framework defects were filled with ZIF-8 crystals, thus stabilizing it in situ through crystallization of ZIF-8 (Figure 10a) [98]. As evidenced by Figure 10b, the GO skeleton defects were filled with ZIF-8 crystals to obtain a GOM with essentially no gap in interlayer spacing compared to the original GOM; thus, it was determined that ZIF-8 grew at the defects rather than between the GO layers. The selective growth of ZIF-8 in the microporous defects conferred mechanical integrity to the laminar skeleton, resulting in stable microstructures while forming selective nano channels that subsequently allowed for fast water transport for more than 180 h without stability-related performance degradation being observed. Zhan et al. prepared NH_2_-POSS@GO hybrid membranes by intercalating aminopropyl isobutyl polyhedral sesquisiloxane (NH_2_-POSS) into the GO interlayer [15]. As indicated in Figure 10c, there were two main typical transport channels in the NH_2_-POSS@GO membrane, including the lateral 2D interlayer channel (type I) and the longitudinal transfer channel (type II), with the longitudinal mass transfer channel provided by the fracture pores around the edges of the GO sheets. POSS was mainly bonded covalently with the carboxyl groups in the slit holes around the edges of the GO lamellae and the epoxy regions on the substrate, and water molecules could flow rapidly through the 2D interlayer channels. POSS particles anchored at the edges of the GO lamellae might aggregate and rearrange in the slit holes, where the POSS cage acted as a fishing net by suturing and compensating for the longitudinal mass transfer channel defects. The mesh size was sufficient enough to allow water penetration but would exclude salt ions. The large longitudinal mass transfer channels of GOMs make it difficult to be efficiently applied in desalination. Shi et al. prepared ultrathin PA-GO membranes with excellent desalination performance using a confined interfacial polymerization method (Figure 10d) [99]. The adsorption of negatively charged GO with oxygen-containing groups towards m-phenylenediamine (MPD), followed by interfacial polymerization of MPD with trimesoyl chloride (TMC) in the void region of the GO layer to obtain small-sized polyamides (PA), allowed the longitudinal mass transfer channels of GOMs to be refined for efficient desalination. The obtained PA-GO membranes showed a high desalination rate of 99.7%, achieving a high permeation rate of 3.0 L m^−2^ h^−1^ bar^−1^ due to their 30 nm thickness and small amount of formed PA. In addition, the PA-GO membranes exhibited good long-term stability, high chemical stability, and low contamination.

Due to the imperfect interlayer stacking of GO, microporous defects (skeleton defects) exist in the entire longitudinal mass transfer channel of GOMs. These microporous defects are the critical limiting factors for the water–solute selectivity of GOMs. Compensating for longitudinal mass transfer channel defects can increase GOM flux without sacrificing layer spacing, and the filling material at the defects can also act as a salt ion barrier, thus improving the salt rejection rate of GOMs. At present, there are many methods to adjust the two-dimensional mass transfer channel, but there are few studies that have focused on compensation of the longitudinal mass transfer channel. The defects of the longitudinal mass transfer channel have a great impact on the salt rejection rate of GOMs and the stability of the membrane structure. It is still a very challenging task to realize a GOM with high selectivity and stability.

#### 4.2.2. Construction of Fast Longitudinal Mass Transfer Channels

Porous graphene has attracted increasing attention in separation applications due to its unique porous structure and the inherent properties of graphene. Theoretical calculations show that, compared to traditional reverse osmosis membranes, nanoporous graphene membranes can significantly improve water flux by several orders of magnitude, making them an excellent prospective membrane material for water purification [22].

**Figure 11:**
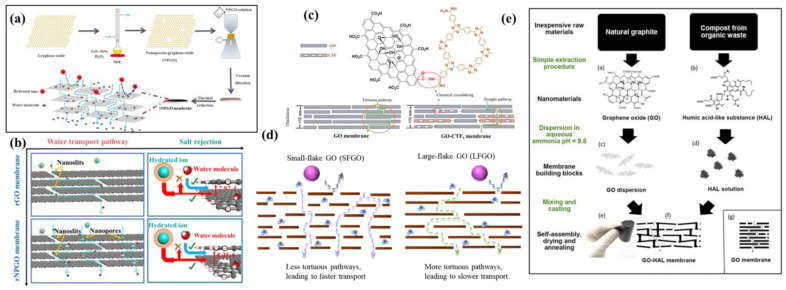
(**a**) Schematic diagram of the preparation process of a reduced nanographene oxide membrane (RNPGO). Reproduced from [100] permission of ACS, 2019. (**b**) Water transport and salt rejection mechanism of RGO and rNPGO membranes. Reproduced from [100] permission of ACS, 2019. (**c**) Difference between GOM and GO-CTF membrane longitudinal mass transfer channels. Reproduced from [101] permission of ACS, 2019. (**d**) Schematic diagram of the transport path of methanol molecules in SFGO and LFGO membranes. Reproduced from [102] permission of AAAS, 2020. (**e**) The preparation process of the GO-HAL membrane and the transport path of water molecules in the GO-HAL membrane. Reproduced from [103] permission of Elsevier, 2017.

The formation of nanopores on the GO layer builds longitudinal mass transfer channels. Li et al. used H_2_O_2_ oxidation to create high-density nanopores on GO nanosheets. Under the same GO coverage, the water flux of the reduced nanoporous GO (rNPGO) membrane was as high as 39.93 ± 0.46 LMH/bar. In contrast, the water flux of the rGO membrane was only 1.53 ± 0.59 LMH/bar (Figure 11a) [100]. GO forms hybridized membranes with covalent organic backbones (COFs) to construct longitudinal mass transfer channels. Khan et al. designed hybrid nanosheets composed of chemically grafted GO and covalent organic backbones (COFs) as building blocks to prepare hybrid nanosheet membranes (Figure 11c) [101]. The covalent triazine skeleton with triazine as a moiety was exfoliated into nanosheets and then reacted with GO to form GO-CTF hybrid nanosheets. The addition of CTF nanosheets had little effect on the interlayer spacing of the GO-CTF membrane; however, the CTF nanosheets provided additional channels through the planar surface, significantly shortening the water transport path. The water flux of the GO-CTF membrane was 226.3 L m^−2^ h ^−1^ bar^−1^, which is more than 12 times higher than that of the pure GOM. In addition, the strong chemical bond between GO and the COF enabled the stability of GO-CTF membranes to be significantly improved. The grafting of porous nanosheets onto non-porous nanosheets to obtain hybridized nanosheets as building blocks has indicated a new way of preparing two-dimensional membranes with promising applications.

The mass transfer pathway of small molecules can be change by modulating the sheet size of GO. Nie et al. prepared two sheet sizes of GOMs and showed that the GOM with a small sheet size had larger permeation rates due to the shorter transverse mass transfer paths [102], which allowed for the rapid transport of small molecules through shorter longitudinal mass transfer paths (Figure 11d).

Short longitudinal mass transfer channels can be created by polymer-induced alignment of GO sheets. Boffa et al. used HAL, a humic acid-like polymer extracted at high yield from composted organic municipal waste [103], to induce the appearance of highly disordered structures in GOMs, thereby increasing their permeability upon thermal stabilization, as shown in Figure 11e.

Regulation of the longitudinal mass transfer channel in the GOM is mainly carried out from two aspects: one involves inorganic particle filling and PA confinement polymerization in the GO layer to compensate for longitudinal defects, and the other involves constructing or changing the longitudinal mass transfer nanochannel through a series of methods (such as drilling on the nanosheet and controlling the size of the nanosheet), with a hybrid membrane formed by mixing the inorganic skeleton with GO nanosheets. From these two aspects, the longitudinal mass transfer channel of the GOM can be effectively regulated, and water permeability can be improved without sacrificing the waste liquid rate, which provides some ideas for the application of GOMs in permeable steam separation. However, in order to be applied in industry and practical operations, a lot of simulations and experiments need to be conducted.

### 4.3. Regulation of GO Surface Wrinkles

Parallel nanochannels formed between GO sheets are the main mass transfer channels due to their easily adjusted size and chemical properties, while longitudinal mass transfer channels between GO sheets have similar properties and have been extensively studied. In addition to these channels, wrinkled forms also exist in two-dimensional membranes, and the structure and potential transport role of this curved channel remains largely unexplored. The results show that the nanowrinkles formed in graphene-based membranes are basically curved shapes with a central height of 2~3 nm and narrow wedge angles formed on both sides [33].

**Figure 12:**
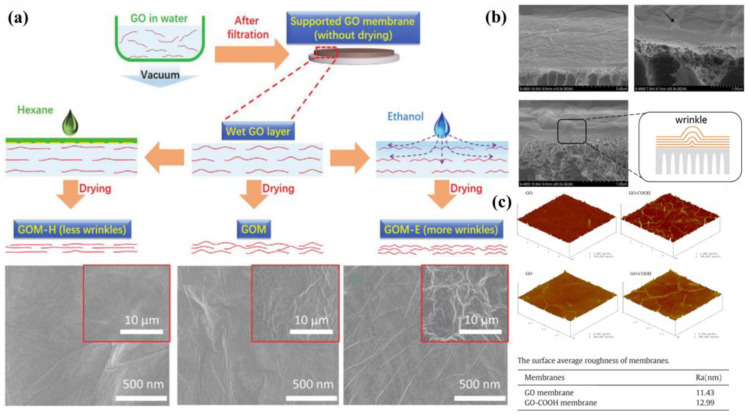
(**a**) Schematic diagram of the process related to wrinkles appearing on GOMs and SEM images of GOMs-H, GOMs, and GOMs-E. Reproduced from [33] permission of Wiley, 2020. (**b**) Detailed morphology of membrane surface wrinkles at different magnifications: 10,000×; 35,000×; 50,000×. Reproduced from [104] permission of Elsevier, 2017. (**c**) AFM images of GO and GO-COOH membranes and their roughness. Reproduced from [104] permission of Elsevier, 2017.

Kang et al. prepared GOMs with different wrinkle densities by adjusting the drying process during film formation using different solvents [33]. The intercepted water between adjacent nanosheets evaporated rapidly (drying step), and insufficient vacuum-induced residual stress release resulted in the formation of wrinkles. They applied ethanol or hexane to the original GOMs to adjust the membrane drying process for wrinkle treatment, and the treated membranes were denoted as GOMsE and GOMsH. Compared to pristine GOMs, GOMsE possessed more nanowrinkles, while GOMsH presented a smoother surface and low fold density (Figure 12a). This difference could be attributed to the fact that the different polarity of the solvent used affects the interaction between water and nanosheets during the drying process, which in turn changes the stacking pattern of GO nanosheets in the dried membranes. The wrinkles of GO membranes can also be increased by adding functional groups to GO membranes, and Yuan et al. produced highly structured GOMs and GO-COOH membranes by using one-step carboxylation through a nucleophilic substitution reaction between the epoxy group on the GO and the amino group of glycine [104]. As the results showed, GO-COOH membranes not only had higher permeability and better desalination rates compared to the pristine GOMs, but also improved surface physicochemical properties, including electronegativity and hydrophilicity. SEM revealed a large number of folds on the surface of the membrane (Figure 12b), and AFM analysis revealed a more detailed structure, where the folds on the surface of the GO-COOH membrane increased and became more pronounced due to the attachment of carboxyl groups. Figure 12c shows AFM 3D surface images of the GOMs and GO-COOH membranes. Compared to GOMs, the number of folds on the surface of the membranes increased significantly, and the surface of GO-COOH membranes showed more obvious “peaks” and higher surface roughness.

In addition to the two-dimensional channels between the GO sheets and the longitudinal mass transfer channels, the wrinkles on the GOM surface are also one of the channels that have been neglected for a long time. In most previous theories and simulation models, GO nanosheets are assumed to be smooth. In fact, as a two-dimensional flexible material, GO nanosheets wrinkle during assembly. Usually, wrinkles that appear in the GO layer turn into additional transmission channels that are more spacious. Researchers have mostly focused on the introduction of extramembrane wrinkles and paid little attention to wrinkles within the GOM. Therefore, there is a lot of room to study how wrinkles are formed and regulated within the GOM, as well as the role of wrinkles in the mass transfer process, which will provide inspiration for the design and preparation of high-throughput wrinkled GOMs in the future.

Compared to other desalination membranes, GOMs have significant advantages in seawater desalination: (1) single-layer GO has a large specific surface area, which, combined with the functional properties of carbon atoms, means that there are many sites for the introduction of surface functional groups that allow for adjustments to GO surface properties [105]; (2) graphene materials have high thermal conductivity of 5000 W m^−1^ K^−1^ [106] (making them ideal for pervaporation desalination based on solar driven heating) and good electrical conductivity (which means they can be used as electrodes for capacitive desalination); (3) GOMs change physicochemical structures through surface modification to reduce adhesion, which also has great prospects in antifouling. Most of the studies included in this review have been associated with improved flux and salt rejection rates due to GOM modification, and there is no doubt that reducing membrane surface contamination will also be an important factor when considering actual desalination in the future. The desalination performance of different GOMs is shown in Table 1 and Table 2.

## 5. Conclusions and Perspectives

GOMs with ultrafast water transport nanochannels have attracted great attention in the field of membrane separation. However, it was worth noting that the wide application of GOMs in membrane technology is still facing several challenges, such as poor membrane stability in aqueous environments and precise regulation of the interlayer spacing of GOMs [111,112]. This review described the mass transfer mechanism of the structure of GO and the preparation methods of GOMs in brief. The regulation of GOM interlayer spacing, longitudinal mass transfer channels, and membrane surface wrinkles was summarized in detail to clarify the relationship between microstructure and desalination performance and provide some new insight into the structural design of high-performance GOMs. It was found that covalent cross-linking, cation intercalation, polymer and inorganic particle intercalation, and the use of MOFs to decorate longitudinal mass transfer channels could effectively improve the stability of GOMs while improving separation performance. However, there are still enormous challenges to be overcome to realize the industrial application of GOMs. (1) GOM stability: The swelling of GOMs in aqueous solutions and the hydration of hydrophilic functional groups on GO with water molecules resulted in an increase in the interlayer repulsion of GO and an increase in interlayer spacing. Therefore, the structure of GOMs in aqueous solutions was easily destroyed by decomposition and separation ability was completely lost. Enhancing hydrophobicity or interlayer interaction without sacrificing interlayer spacing and membrane surface hydrophilicity might be a promising way of improving the swelling resistance of GOMs. (2) Preparation of large-area GOM: GO nanosheets are prone to inhomogeneity during the stacking process due to interlayer rejection, thus forming defects. In the preparation of large-area GOMs, these non-selective defects would be fatal drawbacks affecting the performance of GOMs. There were few studies that provided details about the preparation of large-area GOMs with excellent performance. Liu et al. [113] successfully developed a process for the continuous preparation of large-area ultrathin reduced GO membranes with an area of 30 × 80 cm^2^ and a thickness of few nanometers, which exhibited ideal permeability flux (60.0 kg m^−2^ h^−1^) and an acceptable salt rejection rate (96.0%). It can be seen that although large-area pure GO membranes have good nanofiltration performance, compared to other studies on small-area modified GO membranes, there is still a large space for improvement in terms of flux and retention rate. How to prepare large-area GO membranes with cross-linked structures or particle intercalation and put them into industrial application may be the focus of future research. (3) Improve the permeation flux of GOMs: GOMs are constrained by the trade-off effect. When the salt rejection rate is high, flux is not ideal. It is urgent that a way to break the trade-off effect is found. (4) Potential toxicity of GO: Several groups [114,115,116] reported that even trace doses of GO might also reach the aquatic environment, accumulate, and thus affect biota interactions, which leads to bioaccumulation and acute toxicity in the liver. If GOMs are widely used in desalination, it is reasonably speculated that unstable GOMs would result in residual traces of GO in water under cross-flow scouring with high pressure or temperature. Therefore, the potential toxicity of GO cannot be ignored in the various applications of GOMs, which should be evaluated in detail before industrial application.

Based on the investigations and analysis mentioned above, there are several strategies for the further development of GOM. (1) Development of mixed dimensional GOMs [117]: Graphene oxide (GO) and reduced GO are attracting attention for their unique advantages, such as high specific surface area and mechanical strength, excellent flexibility, ultra-low friction to water, and the applicability of the process to mass production. GO has high levels of oxygen-containing groups, including hydroxyl, carboxyl, and epoxide groups, which can confer covalent interactions, hydrogen bonding, and complexation. Using GO as a platform, the mixed dimensional GOMs were prepared by intercalating nanomaterials into GO. According to the different intercalated molecules, the mixed dimensional GOMs can be divided into three types: 1D/2D membranes (intercalating 1D materials between adjacent GO layers, such as nanofibrils, nanorods, and nanotubes), 0D/2D membranes (intercalating 0D materials between adjacent GO layers, such as ions, molecules, quantum dots, and nanoparticles), and 3D/2D membranes (intercalating 3D materials between adjacent GO layers, such as MOFs, zeolites, and some composite nanomaterials). Different dimensional intercalation materials could be precisely constructed in two-dimensional horizontal mass transfer channels or longitudinal mass transfer channels to build mixed dimensional mass transfer channels that would allow for the selective and rapid transfer of small molecules in multi-dimensional mass transfer channels. (2) Development of bio-inspired materials/GO composite membranes: The large number of biological structures and materials in nature provides important insights for the preparation of GOMs with high water resistance and structural stability, such as the adhesive behavior of proteins in mussels. Lee et al. found that dopamine is capable of forming polymer-like coatings on a variety of substrates through oxidized self-polymerization [118]. The rich oxygen-containing functional groups of GO nanosheets provided the possibility of combining them with bionanomaterials; for instance, dopamine could be coated on the surface of GOMs [119]. Combination with bionic materials will increase the mechanical stability and surface flexibility of GOMs, which will thus provide innovative research ideas and directions for the preparation of high-stability large-area GOMs. (3) Precise construction of pores on the GO planes: The precise construction of sub-nano/nano pores on the surface of GO sheets did not change the interlayer spacing of GOMs, which could allow for the number of longitudinal mass transfer channels to be increased or the length of longitudinal mass transfer channels to be shortened without sacrificing the salt rejection rate of GOMs. This would allow water molecules to pass through GOMs quickly and improve permeation flux. By regulating pore size and the charge around the pore of sub-nano/nano pores, hydrated ions could be effectively trapped and the rejection rate could be improved. (4) Further investigation of the separation mechanism of GOMs: It was generally considered that water molecules permeate through the interconnected nanochannels formed between GO nanosheets and penetrate along tortuous paths mainly on the hydrophobic non-oxidized surface of GO rather than in the hydrophilic oxidized region [120]. The virtually frictionless surface of non-oxidized GO is conducive to the extremely fast flow of water molecules, and size exclusion and/or Donnan exclusion appear to be the dominant sieving mechanisms. The separation mechanism of GO should be further studied based on both molecular dynamics simulation and experimental analysis so as to guide the precise design of high-performance GO membranes [121].

## Figures and Tables

**Table 1:** Desalination performance of GOMs based on NF and RO.

Abbreviation of GO Membrane	Membrane Classification	Applied Pressure	Feed	Operating Temperature	Flux (LMH/Bar)	Rejection %	Reference
HRGO	NF	0.4 MPa	1 g/L Na_2_SO_4_	RT	~1.8	~90	[57]
MRGO	NF	0.4 MPa	1 g/L Na_2_SO_4_	RT	~3	~95	[57]
rNPGO	NF	0.6 MPa	20 Mm Na_2_SO_4_	RT	~39.93	~90	[100]
BPPO/EDA/GO	NF	0.1 MPa	1 g/L Na_2_SO_4_	RT	4.1	56.2	[107]
PEI/GO/PEI	NF	0.5 MPa	1 g/L Mg_2_SO_4_	30 °C	4.2	93.9	[38]
g-C3N4	NF	0.4 MPa	1 g/L Na_2_SO_4_	RT	152	98.9	[13]
PIP-GO	NF	0.27 MPa	0.5 g/L Mg_2_SO_4_	RT	242	90	[108]
GO/phenolic	NF	0.1 MPa	0.5 g/L NaCl	RT	165.6	97	[109]
K-rGO	NF	0.6 MPa	1 g/L NaCl	RT	~1.1	~91	[68]
PA-GO	RO	1 MPa	2 g/L NaCl	RT	3	99.7	[99]

**Table 2:** Desalination performance of GOMs based on PV.

Abbreviation of GO Membrane	Membrane Classification	Feed	Operating Temperature	Flux (kg·m^−2^·h^−1^)	Rejection%	Reference
GO/PAN	PV	2 wt% NaCl	90 °C	65.1	99.8	[2]
Alg-GO-1	PV	3 wt% NaCl	40 °C	3.46	99.95	[16]
A4-GO	PV	3.5 wt% NaCl	75 °C	19.7	99.99	[73]
GOF	PV	3.5 wt% NaCl	90 °C	11.4	99.9	[75]
POSS@GO	PV	3.5 wt% NaCl	80 °C	112.7	99.98	[15]
CDA-GOCM	PV	3.5 wt% NaCl	90 °C	20.1	99.9	[76]
CS/GO MMMs	PV	5 wt% NaCl	81 °C	30	99.99	[110]
GO-PVA	PV	10 wt% NaCl	90 °C	98.0	99.99	[84]
c-GO-PEI 70k	PV	10 wt% NaCl	90 °C	86.0	99.9	[19]
GO-PVAm-Silica	PV	10 wt% NaCl	70 °C	80.2 ± 0.8	99.99	[90]
